# Accurate Nonlinearity and Temperature Compensation Method for Piezoresistive Pressure Sensors Based on Data Generation

**DOI:** 10.3390/s23136167

**Published:** 2023-07-05

**Authors:** Mingxuan Zou, Ye Xu, Jianxiang Jin, Min Chu, Wenjun Huang

**Affiliations:** 1 College of Control Science and Engineering, Zhejiang University, Hangzhou 310027, China; 2 China Petroleum & Chemical Corporation, Beijing 100728, China

**Keywords:** piezoresistive pressure sensor, multidimensional compensation, data generation, mixed polynomial kernel extreme learning machine, aquila optimization, high-accuracy measurement

## Abstract

Piezoresistive pressure sensors exhibit inherent nonlinearity and sensitivity to ambient temperature, requiring multidimensional compensation to achieve accurate measurements. However, recent studies on software compensation mainly focused on developing advanced and intricate algorithms while neglecting the importance of calibration data and the limitation of computing resources. This paper aims to present a novel compensation method which generates more data by learning the calibration process of pressure sensors and uses a larger dataset instead of more complex models to improve the compensation effect. This method is performed by the proposed aquila optimizer optimized mixed polynomial kernel extreme learning machine (AO-MPKELM) algorithm. We conducted a detailed calibration experiment to assess the quality of the generated data and evaluate the performance of the proposed method through ablation analysis. The results demonstrate a high level of consistency between the generated and real data, with a maximum voltage deviation of only 0.71 millivolts. When using a bilinear interpolation algorithm for compensation, extra generated data can help reduce measurement errors by 78.95%, ultimately achieving 0.03% full-scale (FS) accuracy. These findings prove the proposed method is valid for high-accuracy measurements and has superior engineering applicability.

## 1. Introduction

Piezoresistive pressure sensors based on micro-electro-mechanical systems (MEMSs) have been widely used in industrial control [1,2], health monitoring [3,4], automotive applications [5,6], and wearable equipment [7,8]. Diversified application scenarios and working conditions create lots of challenges for pressure sensors. Especially in many industrial fields such as oil refining and chemical production, pressure sensors are usually required to achieve high-accuracy (typically 0.05% FS) and high-stability measurement over a wide range of temperatures (typically from −40 to 85 degrees Celsius). Unfortunately, the inherent nonlinearity of the pressure sensor seems to be unavoidable. Material properties, structural design, manufacturing process, and process bias all contribute to this result. In addition, changes in the measurement environment (mainly temperature) exacerbate the output response error and significantly reduce the measurement accuracy. Therefore, sensor compensation considering both nonlinearity and temperature is essential to obtain high-accuracy measurement results. Previous studies have proposed many compensation methods, which generally can be divided into the hardware self-compensation approach and software algorithm compensation approach.

Hardware self-compensation approaches mitigate temperature drift and nonlinearity error primarily through built-in analog circuits [9,10,11], which are easy to implement and offer good real-time performance. But the built-in circuits are also susceptible to temperature changes, which limits the sensor accuracy. Particularly in some industrial applications requiring a wide temperature range, a 1% full-scale error (FSE) level will still remain after compensation [12]. In conclusion, the difference between the multiple sensor responses and the corresponding compensation hardware characteristic makes it difficult for the hardware compensation approach to attain high-accuracy measurement in batch production.

Software algorithm compensation approaches show strong robustness and demand less manufacturing technology; therefore, they have numerous applications in practical engineering. In essence, this approach is a post-compensation method. First, the pressure sensor needs to be placed in different temperature environments, applying different pressures and recording the response voltage of the sensor. These data about the input–output response relationship of the sensor are called sensor calibration data. Then, based on the calibration dataset, additional compensation algorithms are used to correct the sensor output, such as various numerical calculation methods: look-up table algorithm [13], polynomial interpolation [14], and the hybrid compensation method [15]. Over the past few decades, researchers have concentrated on developing more efficient compensation algorithms, including some machine learning techniques [16,17,18], such as artificial neural networks, support vector machines (SVMs), and extreme learning machines (ELMs). They also used meta-heuristic algorithms [19,20] and ensemble learning methods [21] for temperature and nonlinearity compensation.

However, existing research on software compensation has some problems, for example, ignoring the importance of calibration data and the limitations of computational resources. Calibration data are the basis for sensor compensation and increasing the amount of these data can easily improve the compensation effect. Due to the time-consuming and energy-consuming calibration process, it is not advisable to increase the amount of data by adding testing temperature points and pressure points, as it would bring more costs and reduce the production efficiency of the sensor. In the production process, one must make a trade-off between calibration costs and target accuracy, while selecting the appropriate compensation algorithm as well. Moreover, compensation algorithms are always executed by a microcontroller unit (MCU) with limited computing resources. Compared with traditional numerical fitting methods, machine learning, and intelligent optimization algorithms improve compensation accuracy and induce higher computational complexity at the same time. It forces manufacturers to endure more input and output delay times (even being unable to run) or choose more expensive microcontrollers [22,23].

In this study, we present a novel compensation method to solve the above problems. The feature of this method is that it uses the proposed aquila optimizer optimized mixed polynomial kernel extreme learning machine algorithm to learn the calibration process and generate more data. Then, it uses the bilinear interpolation algorithm for sensor compensation in the dataset containing real calibration data and extra generated data to improve the measurement accuracy. The proposed method can effectively realize high-accuracy measurements in a wide temperature range with a small amount of calibration experiment time. Notably, the training and prediction of the AO-MPKELM model described in this method are implemented by an industrial personal computer. Only the bilinear interpolation is executed by the MCU; thus, the present method has lower complexity and stronger engineering practicability than those studies that use the machine learning method as the compensation algorithm. This study is the first to propose that compensation accuracy can be improved by generating more calibration data (without increasing calibration time), which is highly innovative.

The remainder of this paper is organized as follows. In Section 2, the input–output response of the pressure sensor and calibration compensation processes are theoretically analyzed, and the specific implementation steps of the presented compensation method are described. Section 3 provides the details of the proposed calibration data generation algorithm AO-MPKELM. Section 4 analyzes and discusses the results of the calibration and compensation experiments, and the conclusions are presented in Section 5.

## 2. Principle and Method of Pressure Sensor Compensation

### 2.1. Pressure Sensor Response

**Figure 1:**
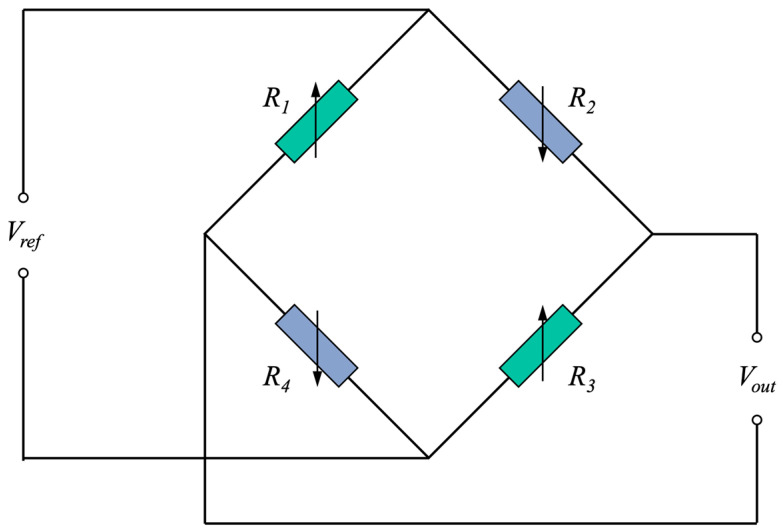
Measurement principle of the piezoresistive pressure sensor.

The piezoresistive pressure sensor is a device that utilizes the piezoresistive effect of silicon. Figure 1 shows the measurement principle of the piezoresistive pressure sensor; it uses four silicon-doped varistors to form a Wheatstone full bridge to convert the input pressure signal to the output electrical signal. The applied pressure causes the varistor to deform, resulting in a change in resistance value and simultaneously affecting the output voltage. In addition, the ambient temperature and the usage time can also affect the sensor characteristics. Silicon is a temperature-sensitive semiconductor, which makes the pressure sensor present different characteristics at different temperatures. If an input signal p is given to a sensor s, its output voltage response $u_{s}$ can be expressed as follows:

(1) $$u_{s}=r_{s}\left(p,T,t\right)+\Delta H_{s}+n_{s},$$

where $r_{s}\left(p,T,t\right)$ denotes the response function of the pressure sensor, p is the applied real pressure, T and t represent current ambient temperature and sensor usage time, respectively, $\Delta H_{s}$ is the uncertain hysteresis caused by the mechanical parts and structure materials, and $n_{s}$ represents the system noise. In this study, the current ambient temperature was measured using an additional temperature sensor.

With the development of manufacturing technology, various system errors and time changes (typically in the scale of years) have less impact on sensor performance; therefore, (1) can be simplified into the following form:

(2) $$u_{s}=r_{s}\left(p,T\right).$$

### 2.2. Calibration and Compensation

In fact, the calibration process of the sensor is the sensor response function (2). The input–output response relationship can be obtained by placing the sensor at different pressures and temperatures. In this process flow, sensor characteristics, measurement range, working temperature zone, and target accuracy should be considered simultaneously when determining the specific number and value of pressure and temperature points. Additionally, the cumulative errors of multiple processes in production will lead to differentiation between the nonlinear characteristics and the piezoresistive effect [22] of each sensor, even for sensors produced in the same batch. So, calibrating each pressure sensor itself is necessary to achieve high-accuracy measurement; that is, each sensor must use its own calibration experiment data for compensation.

Taking sensor s as an example, assuming that $n_{p}$ pressure points and $n_{T}$ temperature points need to be tested, the acquired calibration dataset includes the input–output response of $n_{p}\times n_{T}$ groups. This calibration experiment dataset can be mathematically expressed by

(3) $$S_{C}=\left\{\left(p_{i},T_{j},u_{ij}\right)\right\},\;i=1,\cdots ,n_{p},\;j=1,\cdots ,n_{T}.$$

After obtaining the reference calibration dataset, the voltage response and the temperature value obtained by the additional temperature sensor can be used to compensate for measurement errors. This process can be expressed as follows:

(4) $$\hat{p}=\sigma (u,\;T),$$

where $\hat{p}$ is the compensated pressure (i.e., the measurement result), and σ is called the compensation function [12]. As described in Section 1, many algorithms have been used to fit this function accurately. But the data are considerably more important than the learning model. Thus, more laboratory calibration data are needed to gain more accurate measurement results.

Finally, $p_{f}$ represents the full-scale range of the pressure sensor. The full-scale error of the sensor measurement results can be expressed as:

(5) $$FSE=\frac{\hat{p}-p}{p_{f}}\times 100\%.$$

### 2.3. Implementation Steps of the Proposed Method

**Figure 2:**
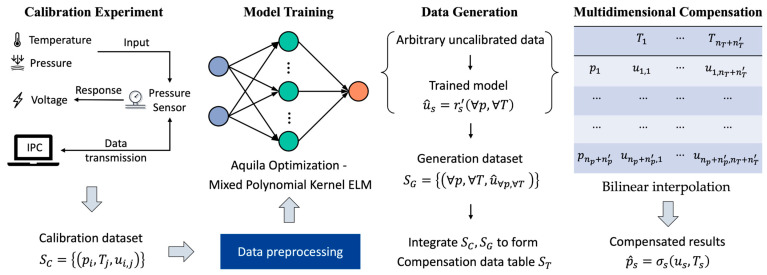
Framework of proposed compensation method based on data generation.

Figure 2 shows the framework of the proposed compensation method based on data generation, which includes the following four steps:

Step 1: Calibration experiment. In this experiment, we need to apply different pressures and temperatures to the sensor, obtain multiple sets of the input–output response relationships, and then transmit the real calibration dataset to an industrial personal computer for data generation.

Step 2: Model training. This step includes data preprocessing, learning the calibration experiment process using the proposed MPKELM algorithm, and the hyperparameter optimization of the learning algorithm using the AO. The detailed theoretical process will be presented in Section 3.

Step 3: Data generation. In the previous stage, we will have learned the calibration process of the sensor. The output response of pressure sensors can be predicted by inputting uncalibrated points at arbitrary temperature and pressure into the trained model. The above process is repeated to obtain the generation dataset $S_{G}$. Finally, $S_{C}$ and $S_{G}$ are integrated to form a compensation data table $S_{T}$, and the values are sorted from small to large. There are now $n_{p}+n_{p}^{\prime }$ pressure points and $n_{T}+n_{T}^{\prime }$ temperature points, where $n_{p}^{\prime }$ and $n_{T}^{\prime }$ are the number of generated pressure and temperature points, respectively.

Step 4: Multidimensional compensation. The compensation data table is sent to the MCU, and a bilinear interpolation algorithm is used to compensate for measurement errors.

## 3. Data Generation Algorithm

### 3.1. Extreme Learning Machine

The ELM [24] is a single hidden layer feedforward neural network (SLFN) learning algorithm. By randomly generating input weights and bias, the output weights of the ELM have a unique least square solution, which can be determined using the Moore–Penrose generalized inverse matrix. Consequently, the training speed of the ELM is significantly faster than traditional SLFNs with a gradient-based learning algorithm. Furthermore, it still retains the powerful nonlinear approximation ability of SLFNs [24,25,26] and performs well in regression and classification problems.

The structure of the ELM is the same as that of traditional SLFNs. Suppose the training set has N arbitrary samples $\left(x_{i},y_{i}\right)$, where $x_{i}={\left[x_{i1},x_{i2},\cdots ,x_{in}\right]}^{T}\in R^{n}$ denotes the inputs, and $y_{i}={\left[y_{i1},y_{i2},\cdots ,y_{im}\right]}^{T}\in R^{m}$ denotes the outputs. The weight w and bias b of each hidden layer node have been randomly generated; therefore, the ELM algorithm only needs to set the number of hidden layer nodes L to calculate the output weight β. Then, the estimated output ${\hat{y}}_{i}$ of the ELM with activation function $g\left(x\right)$ can be expressed by

(6) $${\hat{y}}_{i}=\sum_{j=1}^{L}\beta_{j}g_{j}\left(x_{i}\right)=\sum_{j=1}^{L}\beta_{j}g\left(w_{j}^{T}\cdot x_{i}+b_{j}\right),i=1,\cdots ,N.$$

This equation can be written in matrix form as follows:

(7) $$\hat{Y}=G\beta .$$

(8) $$G={\left[\begin{array}{ccc} g\left(w_{1}^{T}\cdot x_{1}+b_{1}\right) & \cdots & g\left(w_{L}^{T}\cdot x_{1}+b_{L}\right)\\ \vdots & \ddots & \vdots \\ g\left(w_{1}^{T}\cdot x_{N}+b_{1}\right) & \cdots & g\left(w_{L}^{T}\cdot x_{N}+b_{L}\right) \end{array}\right]}_{N\times L}.$$

(9) $$\beta =G^{+}Y={\left[\begin{array}{c} \beta_{1}\\ \vdots \\ \beta_{L} \end{array}\right]}_{L\times m},\;\hat{Y}={\left[\begin{array}{c} y_{1}\\ \vdots \\ y_{N} \end{array}\right]}_{N\times m}.$$

where G is the hidden layer output matrix, $G^{+}$ is the Moore–Penrose generalized inverse matrix of G, β is the unique solution of the output weight calculated by the minimum norm least square method, Y is the expected output, and $\hat{Y}$ is the estimated output.

In [27,28], the orthogonal projection method is applied to solve the Moore–Penrose generalized inverse matrix. Regularization coefficient γ and identity matrix I are introduced to enhance the stability of the ELM. Thus, the output weight β can be expressed as

(10) $$\beta =G^{+}Y=G^{T}{\left(\gamma I+GG^{T}\right)}^{-1}Y.$$

For any sample x, the output function of the ELM is

(11) $$f_{ELM}\left(x\right)=g\left(x\right)\beta .$$

### 3.2. Mixed Polynomial Kernel ELM

The kernel ELM is an extension of the ELM [28] that further improves its generalization ability using a Mercer kernel, such as the Gaussian kernel. If we define the kernel matrix $\Omega =GG^{T}$, with $\Omega_{i,j}=g\left(x_{i}\right)\cdot g{\left(x_{j}\right)}^{T}=\kappa (x_{i},x_{j})$, then (11) can be written as follows:

(12) $$\begin{array}{ll} f_{KELM}\left(x\right) & =g\left(x\right)\beta =g\left(x\right)G^{T}{\left(\gamma I+GG^{T}\right)}^{-1}Y\\ & ={\left[\begin{array}{c} \kappa \left(x,\;x_{1}\right)\\ \vdots \\ \kappa \left(x,x_{N}\right) \end{array}\right]}^{T}{\left(\gamma I+\Omega \right)}^{-1}Y. \end{array}$$

Based on the analysis of the nonlinear output response of the pressure sensor, a mixed polynomial kernel function is designed to accurately simulate the calibration process of the sensor in this study. The mixed polynomial kernel function $\kappa \left(x_{i},x_{j}\right)$ can be defined as follows:

(13) $$\kappa \left(x_{i},x_{j}\right)=\sum_{d=2}^{D}{\theta_{d}\left(x_{i}^{T}x_{j}+1\right)}^{d},$$

where $\theta_{d}$ is the weight of the $d_{th}$ degree polynomial kernel, and D is the highest order of the mixed polynomial kernel function, which needs to be set artificially according to the specific sensor type.

As mentioned above, the output nonlinearity of the sensor is not only affected by the variation of the semiconductor piezoresistive coefficient at different temperatures but also by many other factors in the manufacturing process, which may lead to different output characteristics, even in the same batch. Hence, optimizing the hyperparameters of the proposed MPKELM (including the weights $\theta_{d}$ of each kernel function and regularization coefficient γ) is necessary to improve the robustness of the algorithm.

### 3.3. Parameter Optimization Using Aquila Optimizer

The aquila is a large bird of prey that is active in the northern hemisphere; its agile flight and skillful methods to catch prey make it a top hunter. By observing and imitating the predation process of the aquila, Abualigah et al. [29] proposed the AO algorithm in 2021. Compared with other meta-heuristic algorithms, the AO exhibits considerable superiority in practical engineering problems [29,30,31,32]. The modeling process for the parameter optimization of MPKELM using the AO algorithm is as follows.

#### 3.3.1. Solution Initialization

The AO is a swarm intelligence optimization algorithm. Assume a swarm with population size P, and the problem to be optimized is Q-dimensional; the possible solutions θ of the mixed polynomial kernel parameters to be optimized can be expressed as

(14) $$\theta =\left[\begin{array}{ccc} \theta_{1,1} & \begin{array}{ccc} \cdots & \theta_{1,q} & \cdots \end{array} & \theta_{1,Q}\\ \begin{array}{c} \vdots \\ \theta_{p,1}\\ \vdots \end{array} & \begin{array}{ccc} \ddots & \vdots & \ddots \\ \cdots & \theta_{p,q} & \cdots \\ \ddots & \vdots & \ddots \end{array} & \begin{array}{c} \vdots \\ \theta_{p,Q}\\ \vdots \end{array}\\ \theta_{P,1} & \begin{array}{ccc} \cdots & \theta_{P,q} & \cdots \end{array} & \theta_{P,Q} \end{array}\right],$$

with

(15) $$\theta_{pq}=rand\times \left(UB_{q}-LB_{q}\right)+LB_{q},p=1,\cdots ,P,q=1,\cdots ,Q,$$

where rand is a random number between 0 and 1, and $UB_{q}$ and $LB_{q}$ indicate the $q_{th}$ upper and lower bounds of the parameters to be optimized, respectively.

#### 3.3.2. Global Exploration ${(\theta }_{1})$

When $\tau \leq \frac{2}{3}Τ$, the AO algorithm starts the exploration phase, where τ and Τ denote the current and maximum iteration numbers, respectively. In the first strategy, global exploration, the aquila bird seeks the best prey areas at high altitudes by taking off vertically. The mathematical model of this behavior can be expressed as follows:

(16) $$\theta_{1}\left(\tau +1\right)=\theta_{b}\left(\tau \right)\times \left(1-\frac{\tau }{Τ}\right)+\left(\theta_{M}\left(\tau \right)-\theta_{b}\left(\tau \right)*rand\right),$$

with

(17) $$\theta_{M}\left(\tau \right)=\frac{1}{P}\sum_{p=1}^{P}\theta_{p}\left(\tau \right),\;\forall q=1,\cdots ,Q,$$

where $\theta_{1}\left(\tau +1\right)$ is the solution for the next iteration of τ produced by $\theta_{1}$, $\theta_{b}\left(\tau \right)$ denotes the best solution until the τth iteration, and $\theta_{M}\left(\tau \right)$ represents the average solution of P populations at the τth iteration.

#### 3.3.3. Local Exploration ${(\theta }_{2})$

In the second strategy, local exploration, when the aquila bird has identified a prey area, it circles the target prey, looking for the best time to land and prepare to attack. This behavior is mathematically expressed as follows:

(18) $$\theta_{2}\left(\tau +1\right)=\theta_{b}\left(\tau \right)\times Levy\left(D\right)+\theta_{R}\left(\tau \right)+\left(n-m\right)*rand,$$

where $\theta_{2}\left(\tau +1\right)$ is the solution for the next iteration of τ produced by $\theta_{2}$, $Levy\left(D\right)$ is the levy flight distribution function, which is expressed by (19), and $\theta_{R}\left(\tau \right)$ represents a solution that is randomly selected from P populations at the $\tau^{th}$ iteration.

(19) $$Levy\left(D\right)=\zeta \times \frac{u\times \varepsilon }{{\left|v\right|}^{\frac{1}{\lambda }}},$$

where ζ is a fixed constant 0.01, u and v are random numbers between 0 and 1, and ε is expressed as follows:

(20) $$\varepsilon =\left(\frac{\Gamma \left(1+\lambda \right)\times \sin\left(\frac{\pi \lambda }{2}\right)}{\Gamma \left(\frac{1+\lambda }{2}\right)\times \lambda \times 2^{\left(\frac{\lambda -1}{2}\right)}}\right),$$

where λ is a fixed constant 1.5. m and n in (18) can be calculated using the following formulas:

(21) $$m=r\times \sin\left(\varphi \right)$$

(22) $$n=r\times \cos\left(\varphi \right),$$

with

(23) $$r=r_{1}+U\times Q_{1}$$

(24) $$\varphi =-\omega \times Q_{1}+\frac{3\times \pi }{2},$$

where $r_{1}$ is a random number between 1 and 20, U is a fixed constant 0.00565, $Q_{1}$ represents an integer number between 1 and the optimization problem dimension Q, and ω is a constant fixed at 0.005.

#### 3.3.4. Global Exploitation ${(\theta }_{3})$

When $\tau >\frac{2}{3}Τ$, the AO algorithm starts the exploitation phase. In the third strategy, global exploitation, when the aquila bird has identified a prey area and is ready to land, it descends vertically and launches a tentative attack to see how the prey responds. This behavior can be mathematically expressed as follows:

(25) $$\theta_{3}\left(\tau +1\right)=\left(\theta_{b}\left(\tau \right)-\theta_{M}\left(\tau \right)\right)\times \alpha -rand+\left(\left(UB-LB\right)\times rand+LB\right)\times \delta ,$$

where $\theta_{3}\left(\tau +1\right)$ is the solution for the next iteration of τ produced by $\theta_{3}$, and α and δ are both adjustment parameters that are equal to 0.1.

#### 3.3.5. Local Exploitation ${(\theta }_{4})$

In the fourth strategy, when the aquila bird approaches its prey, it will attack based on the stochastic movement of the prey. The mathematical model of this behavior can be expressed as follows:

(26) $$\theta_{4}\left(\tau +1\right)=QF\times \theta_{b}\left(\tau \right)-F_{1}\times \theta \left(t\right)\times rand-F_{2}\times Levy\left(D\right)+rand\times F_{1},$$

with

(27) $$QF\left(t\right)=\tau^{\frac{2\times rand-1}{{\left(1-Τ\right)}^{2}}}$$

(28) $$F_{1}=2\times rand-1$$

(29) $$F_{2}=2\times \left(1-\frac{\tau }{Τ}\right),$$

where $\theta_{4}\left(\tau +1\right)$ is the solution for the next iteration of τ produced by $\theta_{4}$, QF is a tuning function used to balance different iterative search strategies, which is calculated by (27), $F_{1}$ represents the attack trajectory of the aquila based on the stochastic movement of the prey, and $F_{2}$ denotes the flight slope during the AO algorithm, which decreases from 2 to 0 as the number of iterations increases.

#### 3.3.6. Performance Measurement

In the parameter optimization process of MPKELM, we use the root mean square error (RMSE) for performance measurement. The fitness function can be expressed as

(30) $$E=\sqrt{\frac{1}{m}\sum_{i=1}^{m}{\left({\hat{y}}_{i}-y_{i}\right)}^{2},}$$

where ${\hat{y}}_{i}$ is the predicted value of the MPKELM, $y_{i}$ is the true value, and m represents the number of samples contained in the training set.

#### 3.3.7. Parameter Optimization Flow

**Figure 3:**
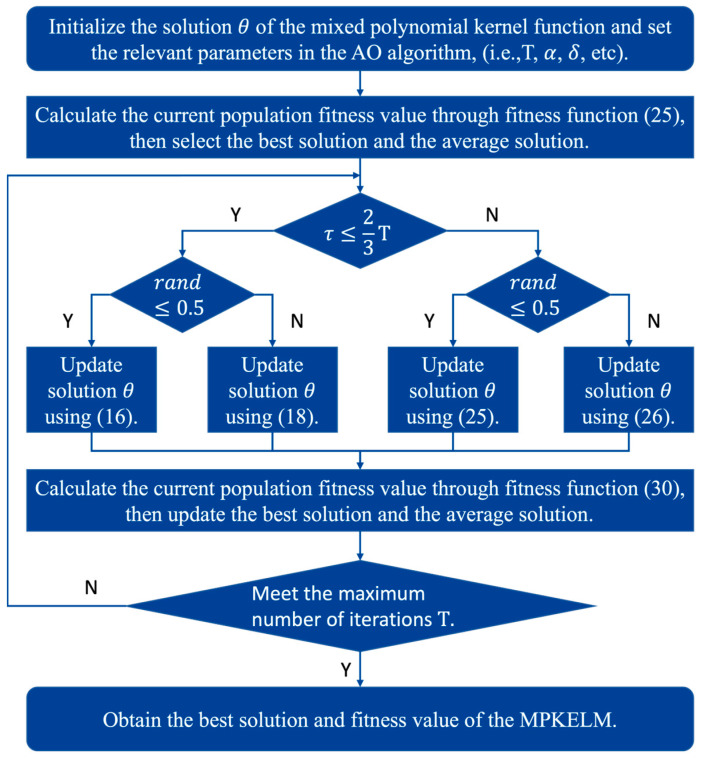
Parameter optimization flow of MPKELM using the AO algorithm.

Figure 3 shows the optimization flow of the MPKELM using the AO algorithm, and the specific process can be summarized in the following four steps:

Step 1: (15) is used to initialize the candidate solutions θ of the mixed polynomial kernel function to start the improvement procedure, and the relevant parameters are set in the AO algorithm, such as the maximum number of iterations Τ and the adjustment parameters α and δ.

Step 2: The current population fitness value is calculated using fitness function (30). Then, the best solution and average solution are selected based on the calculation results.

Step 3: At this step, the iteration begins. When $\tau \leq \frac{2}{3}Τ$, the AO algorithm enters the exploration phase and randomly selects between global exploration and local exploration to update the best solution. When $\tau >\frac{2}{3}Τ$, the AO algorithm enters the exploitation phase and randomly selects between global exploitation and local exploitation to update the best solution.

Step 4: When the current number of iterations τ meets the maximum number of iterations Τ, the optimization process ends, and the optimal parameter solution and fitness value of the MPKELM are output; otherwise, Step 3 is repeated.

## 4. Analysis of Experiments and Results

**Figure 4:**
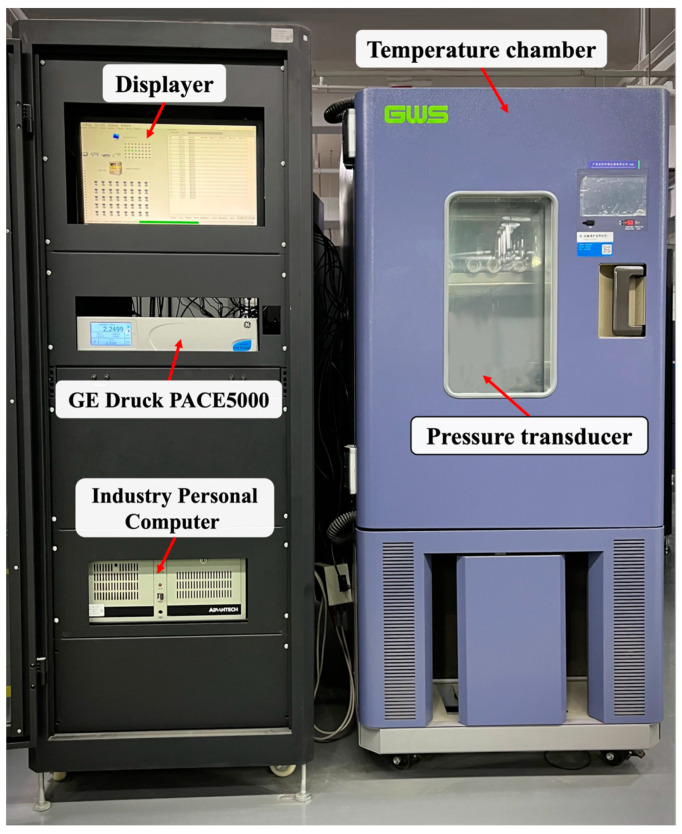
Actual calibration experiment system.

The silicon piezoresistive differential pressure sensor selected in the experiment has a measurement range of −250 to +250 kPa and an operating temperature of −40 to +85 °C. We use a modular pressure controller (GE Druck PACE5000, Baker Hughes Company, Houston, TX, USA) to control the pressure precisely. The accuracy of this device can reach 0.001% FS (including nonlinearity, hysteresis, repeatability, and temperature effects over the calibrated temperature range) within the required working conditions. The experimental temperature environment is determined by a high–low temperature test chamber (ET0440, Guangzhou-GWS Environmental Equipment Company, Guangzhou, China). The set temperature of this equipment ranges from −40 to +150 °C, and the ambient temperature fluctuation is less than 0.5 °C. Figure 4 shows the actual calibration experiment system, where the pressure transducer is the device packaging the pressure sensor and the additional temperature sensor described in Section 2.

First, we carry out a detailed calibration of the sensor equipment to analyze the nonlinearity and temperature drift characteristics without compensation. The pressure calibration points are selected in an arithmetic sequence with a range of −250 to 250 kPa, tested every 31.25 kPa for a total of 17 pressure points. Temperature calibration points are also selected in an arithmetic sequence with a range of −40 to 85 °C, tested every 5 °C for a total of 26 temperature points, and 442 groups of data are finally recorded. A low-noise 24-bit analog-to-digital converter (AD7799) collects the output voltage and transfers the data to the industrial personal computer. Each voltage data point is collected and averaged several times after stability to reduce the measurement uncertainty (the RMS noise of this device is approximately 27 nV).

After collecting the real calibration data, the input–output relationship of the sensor is considered as a simple first-order linear function to obtain the response deviation without compensation. Taking two points, 250 kPa and −250 kPa at room temperature (25 °C), as the benchmark, the uncompensated measurement error can be obtained through calculation.

**Figure 5:**
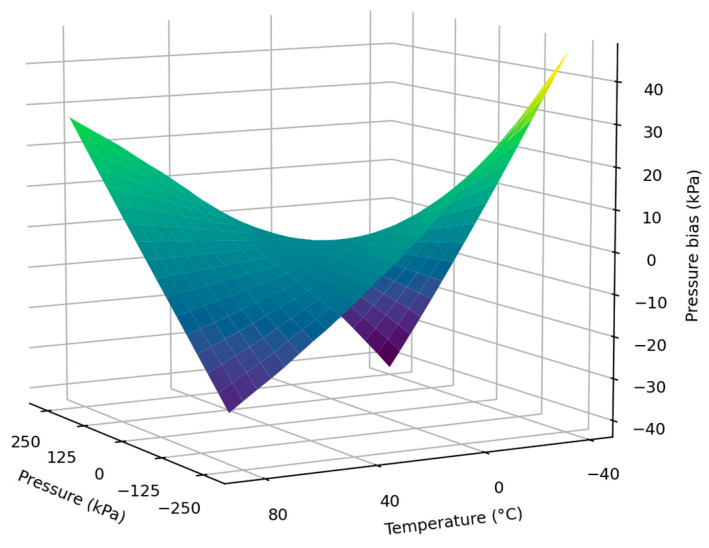
Pressure bias surface before compensation.

Figure 5 is the pressure bias surface before compensation, from which we can find that the measurement error of the silicon piezoresistive pressure sensor increases with the applied pressure increase. This is due to the inherent nonlinear effect between the change in resistance of the silicon-doped resistor and the applied pressure, which becomes more obvious when the input signal varies widely. Meanwhile, as the deviation between ambient temperature and the room temperature (25 °C) increases, the measurement error increases, which is related to the changes in temperature-induced sensitivity. When the sensor is subjected to positive pressure, the measured pressure rises at high temperature and falls at low temperature, and the opposite is true when subjected to negative pressure. The rising temperature decreases semiconductor silicon resistivity, thus reducing sensor sensitivity. Without compensation, the maximum error of this pressure sensor occurs at (−250 kPa, −40 °C) and (250 kPa, −40 °C), which is 46.92 kPa and −42.07 kPa, respectively. We can use (5) to calculate the FSE of these two points as 9.38% and −8.41%, respectively. The results of the experiments demonstrate that the output of the silicon piezoresistive pressure sensor exhibits some nonlinearity and is affected by temperature drift significantly. Consequently, the measurement error of this sensor is considerable without compensation and cannot meet the demands of engineering and scientific applications.

### 4.1. Calibration Experiment

Next, we compare the real calibration data and the generated data to verify the effectiveness of the proposed method. In this experiment, we select five temperature points, −40, −10, 25, 50, and 85 °C, and nine pressure points, −250, −187.5, −125, −62.5, 0, 62.5, 125, 187.5, and 250 kPa, as the calibration dataset for algorithm training, with a total 45 groups of input–output relationships. The remaining 397 groups of experimental data will serve as the test set and will be compared with the generated data to measure the performance of the proposed calibration generation method. Actually, in the batch application of this method, the quality of the generated data does not need to be verified; only the correlation points of the training dataset must be tested. The distribution of the training data is relatively uniform and centralized; therefore, we use (26), the min–max normalization, to normalize different input features $x_{i}$ to the range of [0, 1] to eliminate the influence of data units and improve the convergence speed and accuracy of the model:

(31) $$x_{i}=\frac{x_{i}-min(x_{i})}{\max\left(x_{i}\right)-min(x_{i})}.$$

In the proposed algorithm, the highest order D of the mixed polynomial kernel function is a parameter that needs to be artificially set to better simulate the calibration process of the sensor.

In Table 1, we compare the performance of the AO-MPKELM models under different highest mixing orders, including the RMSE and mean absolute error (MAE) of the training set and test set, as well as the time required by the algorithm. When D=4, the model exhibits the best performance in the test set, and its RMSE and MAE are 1.66 × 10^−4^ and 1.23 × 10^−4^, respectively. When D=5, the model exhibits the highest accuracy on the training set; however, its performance on the test set is inferior to that of the fourth-order AO-MPKELM, which indicates that the introduction of the fifth-order polynomial kernel function has caused certain overfitting problems in the model.

Owing to limited data, the optimization process of the AO is conducted on the training set. The larger the value of D, the more likely overfitting will occur; thus, selecting the correct parameter D is important in the proposed method. Both theoretical and experimental results show that when the model becomes more complex (D increases), the running time of the algorithm will also increase. However, compared with a calibration time of tens of hours, a difference in calculation time of a few seconds between different D levels can be ignored. All the above experiments were conducted using the Python3.7 environment, and the test device used a 10th-generation Intel Core i5 processor and 16 GB memory. There is no doubt that the commonly used industrial computer can meet the above calculation load.

Table 2 shows the set hyperparameter range and optimization results of the AO under different highest mixing orders. In this study, the population number and iteration times of the AO are set at 15 and 20, respectively.

In practical application, we only tested the data of the training set and did not know the real voltage value of the test set; thus, we could not select the hyperparameters according to the results of the test set. Therefore, in the subsequent analysis and experiment, we adopted the AO-MPKELM model (D=5), which performed best on the training set, to express the application effect in the actual project objectively.

**Figure 6:**
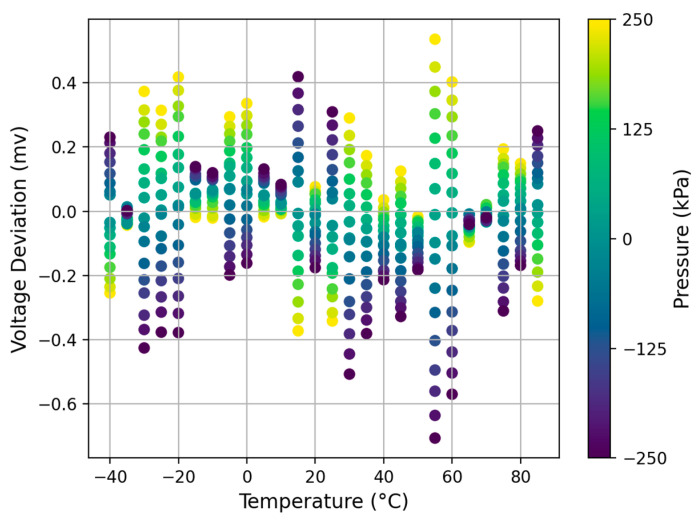
Voltage deviation between generated and real values at each temperature and pressure point.

Figure 6 shows the voltage deviation between generated and real values at each temperature and pressure point. In most cases, the voltage deviation is less than 0.5 mV, and its maximum deviation is 0.71 mV under all working conditions. Through several calibration experiments, we observed millivolt voltage deviations between calibration data in the same working conditions under the influence of superimposed errors by the GE Druck PACE5000, temperature chamber, and pressure sensor. It proves the authenticity and consistency of the generated data and indicates that we can correctly simulate the calibration process of the pressure sensor by modeling its input–output response relationship to generate more calibration data.

### 4.2. Compensation Experiment

After verifying the quality of the generated data, we conducted a series of ablation and comparison experiments to demonstrate the superiority of the proposed method. A variety of compensation algorithms were selected for comparison in the real calibration dataset, including bilinear interpolation (BI), polynomial regression (PR), back-propagation neural networks (BPNNs), support vector machines (SVMs), and kernel extreme learning machine (KELM), all of which have better compensation effects in this field and are currently more suitable for implementation in microprocessors. Although the above algorithms can achieve online compensation, the training process of machine learning still consumes lots of resources, and the BI is undoubtedly the most convenient and fastest algorithm for practical applications. Therefore, in the proposed compensation method based on data generation, BI is adopted as the multidimensional compensation algorithm, and the compensation dataset contains both generated data by AO-MPKELM and real calibration data. We use DG-BI as an abbreviation for this method; the DG represents the data generation process using AO-MPKELM, and BI represents the final compensation algorithms. Some setting parameters of these algorithms are shown in Table 3.

**Figure 7:**
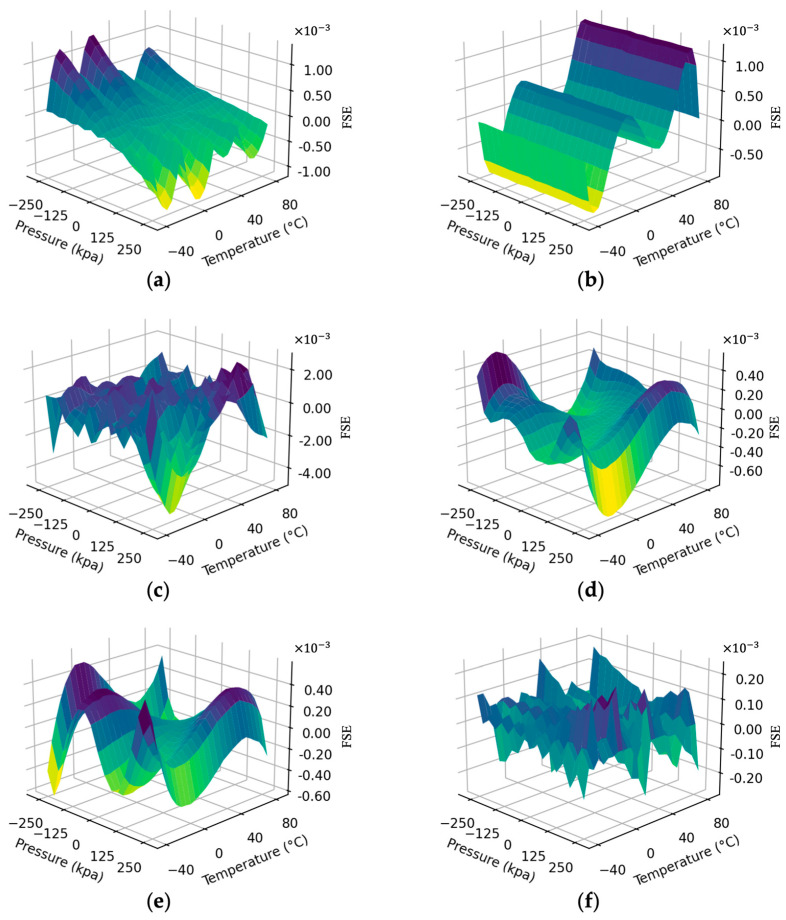
Full-scale error surfaces after multidimensional compensation using different methods: (**a**) bilinear interpolation, (**b**) polynomial regression, (**c**) back-propagation neural network, (**d**) support vector machines, (**e**) kernel extreme learning machine, (**f**) proposed DG-BI method.

Table 4 shows the compensation results of different compensation methods, including the maximum positive full-scale error, maximum negative full-scale error, average full-scale error (absolute value), and standard deviation. Detailed compensation results of different algorithms under different temperatures and pressures are presented in Figure 7. The full-scale error surface of bilinear interpolation is smoother than that of the polynomial regression algorithm, with a smaller average FSE and standard deviation, and the maximum error occurs at a room temperature of 25 °C. The polynomial regression performs poorly at 60 to 80 °C, and the excessively high-dimensional nature caused overfitting in the high-temperature region, so its maximum full-scale error reached 0.123%. As the most popular regression model in recent years, BP has been proven to have a strong generalization ability in various tasks. But in this experiment, the performance is not satisfactory. Insufficient training data hindered us in obtaining optimal weights and bias through gradient descent. The two machine learning methods suitable for small data (SVM and KELM) are both based on the kernel function theory and thus perform similarly, outperforming the models mentioned above. However, there is still nearly 0.06% FSE under extreme working conditions. The proposed DG-BI exhibits the most robust performance and provides the most stable compensation effect over the full-scale and temperature region. Compared to the control group for the ablation experiment that used the BI algorithm only in the real calibration dataset, its measurement error is reduced by 78.95%, which proves the correctness and superiority of the presented theory in this paper.

In this measurement system, the achievable accuracy after compensation is about 0.03% FS, which is better than other methods. The maximum positive full-range error and maximum negative full-range error of the sensor are 0.024% and −0.028%, respectively. Contrasted with the corresponding values before compensation, the errors are reduced to 0.25% and 0.33%. These findings verify that the compensation method in this study can significantly decrease the measurement bias. Moreover, the complexity of this method is lower than that of BP and other learning algorithms; therefore, this method is conducive to achieving high-accuracy compensation in practical engineering applications.

## 5. Conclusions

In this study, we presented a novel multidimensional compensation method, DG-BI, for piezoresistive pressure sensors, which combined the AO and proposed MPKELM to generate more calibration data and used BI for compensation to improve measurement accuracy. Experimental results show that the AO-MPEKLM accurately simulated the calibration process of the pressure sensor and can further improve the compensation effect by using additionally generating high-quality data. The maximum voltage deviation between generated data and actual calibration data is 0.71 mV, and the full-scale error after compensation is 0.028%, only 0.33% of that before compensation. Through the ablation experiment, we found that the measurement accuracy was improved by nearly five times compared to that using only the real calibration data and BI algorithm. This compensation method has a guiding effect on existing compensation research. The high-accuracy measurement can be achieved by using more generated data rather than complex compensation algorithms, which makes software compensation more suitable for engineering applications.

## Figures and Tables

**Table 1:** Performance of the AO-MPKELM models under different highest mixing orders.

D	RMSE		MAE		Time (s)
	Training	Testing	Training	Testing	
2	4.78 × 10^−3^	4.08 × 10^−3^	3.62 × 10^−3^	3.16 × 10^−3^	0.68
3	4.87 × 10^−4^	4.63 × 10^−4^	3.98 × 10^−4^	3.48 × 10^−4^	0.78
4	8.76 × 10^−5^	1.66 × 10^−4^	6.26 × 10^−5^	1.23 × 10^−4^	0.97
5	6.54 × 10^−5^	1.80 × 10^−4^	4.81 × 10^−5^	1.34 × 10^−4^	1.16

**Table 2:** The set hyperparameter range and optimization results of the AO under different highest mixing orders.

Parameter	Range	Result			
		D = 2	D = 3	D = 4	D = 5
γ	[1 × 10^3^, 1 × 10^7^]	7.52 × 10^5^	2.90 × 10^5^	1.91 × 10^4^	3.80 × 10^3^
$\theta_{2}$	[1, 1 × 10^2^]	92.31	6.88	21.86	57.16
$\theta_{3}$	[1, 1 × 10^2^]	0	70.01	13.14	27.49
$\theta_{4}$	[1, 1 × 10^2^]	0	0	27.52	64.79
$\theta_{5}$	[1, 1 × 10^2^]	0	0	0	37.57

**Table 3:** The setting parameters of different compensation algorithms.

Algorithm	Parameter	Value
PR	Highest order	5
BP	Hidden layer number	4
	Optimizer	Adam
	Learning rate	$1\;\times \;10^{-3}$
SVM	Penalty parameter C	$1\;\times \;10^{6}$
	Tolerance	$1\;\times \;10^{-3}$
	Epsilon	$1\;\times \;10^{-3}$
KELM	Penalty parameter C	$1\;\times \;10^{6}$
	Variance σ of gaussian kernel	1

**Table 4:** Compensation results of different methods.

Method	Max Positive FSE	Max Negative FSE	Average	Std
BI	1.33 × 10^−3^	−1.15 × 10^−3^	3.36 × 10^−4^	4.45 × 10^−4^
PR	1.23 × 10^−3^	−9.32 × 10^−4^	4.39 × 10^−4^	5.61 × 10^−4^
BPNN	2.82 × 10^−3^	−4.93 × 10^−3^	8.72 × 10^−4^	1.17 × 10^−3^
SVM	5.48 × 10^−4^	−7.82 × 10^−4^	1.77 × 10^−4^	2.05 × 10^−4^
KELM	5.79 × 10^−4^	−6.11 × 10^−4^	1.83 × 10^−4^	2.25 × 10^−4^
DG-BI	***2.36* × *10^−4^***	***−2.80* × *10^−4^***	***5.90* × *10^−5^***	***7.81* × *10^−5^***

## Data Availability

Not applicable.

## References

[1] Song P., Ma Z., Ma J., Yang L., Wei J., Zhao Y., Zhang M., Yang F., Wang X. (2020). Recent Progress of Miniature MEMS Pressure Sensors. Micromachines.

[2] Jena S., Gupta A. (2021). Review on Pressure Sensors: A Perspective from Mechanical to Micro-Electro-Mechanical Systems. Sens. Rev..

[3] Xu T., Wang H., Xia Y., Zhao Z., Huang M., Wang J., Zhao L., Zhao Y., Jiang Z. (2017). Piezoresistive Pressure Sensor with High Sensitivity for Medical Application Using Peninsula-Island Structure. Front. Mech. Eng..

[4] Kang K., Park J., Kim K., Yu K.J. (2021). Recent Developments of Emerging Inorganic, Metal and Carbon-Based Nanomaterials for Pressure Sensors and Their Healthcare Monitoring Applications. Nano Res..

[5] Qian J., Kim D.-S., Lee D.-W. (2018). On-Vehicle Triboelectric Nanogenerator Enabled Self-Powered Sensor for Tire Pressure Monitoring. Nano Energy.

[6] Soy H., Toy İ. (2021). Design and Implementation of Smart Pressure Sensor for Automotive Applications. Measurement.

[7] Li W., Lu W., Sha X., Xing H., Lou J., Sun H., Zhao Y. (2022). Wearable Gait Recognition Systems Based on MEMS Pressure and Inertial Sensors: A Review. IEEE Sens. J..

[8] Cui X., Huang F., Zhang X., Song P., Zheng H., Chevali V., Wang H., Xu Z. (2022). Flexible Pressure Sensors via Engineering Microstructures for Wearable Human-Machine Interaction and Health Monitoring Applications. iScience.

[9] Wang L., Zhu R., Li G. (2019). Temperature and strain compensation for flexible sensors based on thermosensation. ACS Appl. Mater. Interfaces.

[10] Aryafar M., Hamedi M., Ganjeh M.M. (2015). A Novel Temperature Compensated Piezoresistive Pressure Sensor. Measurement.

[11] Devi R., Gill S.S. (2022). Performance Investigation of Carbon Nanotube Based Temperature Compensated Piezoresistive Pressure Sensor. Silicon.

[12] Pieniazek J., Ciecinski P. (2020). Temperature and Nonlinearity Compensation of Pressure Sensor with Common Sensors Response. IEEE Trans. Instrum. Meas..

[13] Altinoz B., Unsal D. Look up Table Implementation for IMU Error Compensation Algorithm. Proceedings of the 2014 IEEE/ION Position, Location and Navigation Symposium (PLANS 2014).

[14] Ali I., Asif M., Shehzad K., Rehman M.R.U., Kim D.G., Rikan B.S., Pu Y., Yoo S.S., Lee K.-Y. (2020). A Highly Accurate, Polynomial-Based Digital Temperature Compensation for Piezoresistive Pressure Sensor in 180 nm CMOS Technology. Sensors.

[15] Guo Z., Lu C., Wang Y., Liu D., Huang M., Li X. (2017). Design and Experimental Research of a Temperature Compensation System for Silicon-on-Sapphire Pressure Sensors. IEEE Sens. J..

[16] Kayed M.O., Balbola A.A., Lou E., Moussa W.A. (2020). Hybrid Smart Temperature Compensation System for Piezoresistive 3D Stress Sensors. IEEE Sens. J..

[17] Ma Z., Wang G., Rui X., Yang F., Wang Y. (2019). Temperature Compensation of a PVDF Stress Sensor and Its Application in the Test of Gun Propellant Charge Compression Stress. Smart Mater. Struct..

[18] Li J., Hu G., Zhou Y., Zou C., Peng W., Alam J. (2017). SM Study on temperature and synthetic compensation of piezo-resistive differential pressure sensors by coupled simulated annealing and simplex optimized kernel extreme learning machine. Sensors.

[19] Ruan Y., Yuan L., Yuan W., He Y., Lu L. (2021). Temperature Compensation and Pressure Bias Estimation for Piezoresistive Pressure Sensor Based on Machine Learning Approach. IEEE Trans. Instrum. Meas..

[20] Liang H., Chen H., Lu Y. (2019). Research on Sensor Error Compensation of Comprehensive Logging Unit Based on Machine Learning. J. Intell. Fuzzy Syst..

[21] Li J., Zhang C., Zhang X., He H., Liu W., Chen C. (2020). Temperature compensation of piezo-resistive pressure sensor utilizing ensemble AMPSO-SVR based on improved AdaBoost. RT. IEEE Access.

[22] Barlian A.A., Park W.-T., Mallon J.R., Rastegar A.J., Pruitt B.L. (2009). Review: Semiconductor Piezoresistance for Microsystems. Proc. IEEE.

[23] dos Santos Pereira R., Cima C.A. (2021). Thermal Compensation Method for Piezoresistive Pressure Transducer. IEEE Trans. Instrum. Meas..

[24] Huang G.-B., Zhu Q.-Y., Siew C.-K. (2006). Extreme Learning Machine: Theory and Applications. Neurocomputing.

[25] Zheng Y., Chen B., Wang S., Wang W., Qin W. (2022). Mixture Correntropy-Based Kernel Extreme Learning Machines. IEEE Trans. Neural Netw. Learn. Syst..

[26] Shi X., Kang Q., An J., Zhou M. (2022). Novel L1 Regularized Extreme Learning Machine for Soft-Sensing of an Industrial Process. IEEE Trans. Ind. Inf..

[27] Deng W., Zheng Q., Chen L. Regularized Extreme Learning Machine. Proceedings of the 2009 IEEE Symposium on Computational Intelligence and Data Mining.

[28] Huang G.B., Zhou H., Ding X., Zhang R. (2012). Extreme Learning Machine for Regression and Multiclass Classification. IEEE Trans. Syst. Man Cybern. B.

[29] Abualigah L., Yousri D., Abd Elaziz M., Ewees A.A., Al-qaness M.A.A., Gandomi A.H. (2021). Aquila Optimizer: A Novel Meta-Heuristic Optimization Algorithm. Comput. Ind. Eng..

[30] Abd Elaziz M., Dahou A., Alsaleh N.A., Elsheikh A.H., Saba A.I., Ahmadein M. (2021). Boosting COVID-19 Image Classification Using MobileNetV3 and Aquila Optimizer Algorithm. Entropy.

[31] Guo Z., Yang B., Han Y., He T., He P., Meng X., He X. (2022). Optimal PID Tuning of PLL for PV Inverter Based on Aquila Optimizer. Front. Energy Res..

[32] Wang S., Ma J., Li W., Khayatnezhad M., Rouyendegh B.D. (2022). An Optimal Configuration for Hybrid SOFC, Gas Turbine, and Proton Exchange Membrane Electrolyzer Using a Developed Aquila Optimizer. Int. J. Hydrog. Energy.

